# Unique Variant Spectrum in a Jordanian Cohort with Inherited Retinal Dystrophies

**DOI:** 10.3390/genes12040593

**Published:** 2021-04-19

**Authors:** Bilal Azab, Zain Dardas, Dunia Aburizeg, Muawyah Al-Bdour, Mohammed Abu-Ameerh, Tareq Saleh, Raghda Barham, Ranad Maswadi, Nidaa A Ababneh, Mohammad Alsalem, Hana Zouk, Sami Amr, Abdalla Awidi

**Affiliations:** 1 Department of Human and Molecular Genetics, School of Medicine, Virginia Commonwealth University, Richmond, VA 23298-0565, USA; 2 Department of Pathology and Microbiology and Forensic Medicine, School of Medicine, The University of Jordan, Amman 11942, Jordan; zain.dardas@bcm.edu (Z.D.); dunia.aburizeg@gmail.com (D.A.); 3 Department of Molecular and Human Genetics, Baylor College of Medicine, Houston, TX 77030, USA; 4 Department of Ophthalmology, Jordan University Hospital, The University of Jordan, Amman 11942, Jordan; bdourjo@yahoo.com (M.A.-B.); mohammd_73@yahoo.com (M.A.-A.); 5 Department of Basic Medical Sciences, Faculty of Medicine, The Hashemite University, Zarqa 13115, Jordan; tareq@hu.edu.jo; 6 Cell Therapy Center, The University of Jordan, Amman 11942, Jordan; raghda.barham@gmail.com (R.B.); nidaaanwar@gmail.com (N.A.A.); 7 Department of Ophthalmology, Guy’s and St Thomas’ NHS Foundation Trust, London SE1 7EH, UK; Ranad.maswadi@gstt.nhs.uk; 8 Department of Anatomy and Histology, School of Medicine, The University of Jordan, Amman 11942, Jordan; dralsalem@gmail.com; 9 Laboratory for Molecular Medicine, Partners HealthCare Personalized Medicine, Cambridge, MA 02139, USA; hzouk@bwh.harvard.edu; 10 Department of Pathology, Massachusetts General Hospital/Harvard Medical School, Boston, MA 02114, USA; 11 Department of Pathology, Brigham and Women’s Hospital, Harvard Medical School, Boston, MA 02115, USA; samr@bwh.harvard.edu

**Keywords:** inherited retinal dystrophy (IRD), retinitis pigmentosa (RP), whole exome sequencing, retinal genetic testing, unique phenotypes

## Abstract

Whole Exome Sequencing (WES) is a powerful approach for detecting sequence variations in the human genome. The aim of this study was to investigate the genetic defects in Jordanian patients with inherited retinal dystrophies (IRDs) using WES. WES was performed on proband patients’ DNA samples from 55 Jordanian families. Sanger sequencing was used for validation and segregation analysis of the detected, potential disease-causing variants (DCVs). Thirty-five putatively causative variants (6 novel and 29 known) in 21 IRD-associated genes were identified in 71% of probands (39 of the 55 families). Three families showed phenotypes different from the typically reported clinical findings associated with the causative genes. To our knowledge, this is the largest genetic analysis of IRDs in the Jordanian population to date. Our study also confirms that WES is a powerful tool for the molecular diagnosis of IRDs in large patient cohorts.

## 1. Introduction

Inherited retinal dystrophies (IRDs) are a group of diverse hereditary disorders collectively characterized by progressive retinal deterioration [1,2]. IRDs are a primary cause of vision impairment and blindness at different ages, affecting more than two million people worldwide [3]. IRDs exhibit varied clinical presentations and are often classified into two categories: non-syndromic IRDs, such as retinitis pigmentosa (RP), cone or cone-rod dystrophy (CD-CRD) and Leber congenital amaurosis (LCA), and syndromic IRDs, including Bardet–Biedl (BBS), Joubert, Senior-Loken and Usher syndromes [1,4]. IRDs are also characterized by pronounced genetic heterogeneity, with more than 250 genes attributed to the disease (Retina Information Network https://sph.uth.edu/retnet/, accessed on 15 April 2021)

Different variants in a single IRD gene may lead to distinct clinical presentations, including those identified in intra-familial cases [5,6]. The clinical picture of IRDs is largely dependent on the pathogenetic processes leading to retinal damage. For example, RP, the most widespread IRD with a worldwide prevalence of 1 in 3500 to 5000 [7], initially presents with night blindness, typically manifesting in childhood/adolescence, which later deteriorates into loss of peripheral vision. Conversely, CD-CRD degeneration develops initially in the cones followed by the rods [8,9,10], and therefore, its primary presenting symptom is reduced visual acuity and loss of sensitivity in the central visual field, followed by night blindness and loss of peripheral vision [11,12]. However, there is a considerable clinical overlap between RP and CRD, such that in advanced cases, it is not feasible to generate a definitive clinical diagnosis of each disorder. This diagnostic challenge has invited for the liberal utilization of genetic diagnostic approaches [13,14]. Fortunately, the identification of disease-causing variants (DCVs) in familial and sporadic cases of IRDs has been remarkably enhanced by the implementation of next-generation sequencing (NGS) technologies in the genetic diagnostic settings [13,15].

The population of Jordan has remarkably increased from less than a million inhabitants in the 1950s [16] to more than 10 million people in 2021, according to Jordan’s Department of Statistics latest estimations (http://dosweb.dos.gov.jo/, accessed on 15 April 2021). Furthermore, the indigenous population of Jordan consisted historically of Bedouin pastoralists and ancient urbanites [16,17]; however, currently, Jordan is a habitat for diverse ethnic backgrounds, predominantly consisting of Arabs in addition to other ethnic minorities such as Armenians, Circassians and Chechens [18]. Furthermore, consanguineous marriage rates are higher among Jordanians, which has been linked to higher rates of recessive genetic disorders [16]. Unfortunately, neither the prevalence rate of IRDs nor a comprehensive, cohort-wide variant analysis of IRDs have been previously reported in Jordan. In this study, we employed whole exome sequencing (WES) in 55 recruited families suffering from IRDs throughout Jordan, to further delineate the genetic etiologies of IRDs and to further contribute to understanding the variant spectrum for this group of disorders. 

## 2. Materials and Methods

### 2.1. Study Subjects

A group of 87 Jordanian patients from 55 unrelated families suffering from IRD were enrolled in this study, who were recruited from different geographical regions across the country. Patients underwent a standard ophthalmological examination, including BCVA using standard Snellen charts, slit-lamp biomicroscopy (Haag-Streit BM 900, Koeniz, Switzerland) and Optical Coherence Tomography (OCT) (Optovue RTVue, Fremont, CA, USA). IRD diagnosis was made by specialized ophthalmologists. This study was approved by the Institutional Review Board committee of the Cell Therapy Center, Amman, Jordan (protocol code 1/2014, 19 August 2014). Peripheral blood samples were collected from patients as well as their available informative relatives for DNA extraction. Written informed consent that adhered to the tenets of the declaration of Helsinki was obtained from all participants or from a parent and/or legal guardian for participants under the age of 18 years old. All methods were carried out in accordance with the approved guidelines.

### 2.2. Exome Sequencing and Data Analysis

WES was performed on DNA samples of 55 proband patients by the laboratory for molecular medicine (LMM), Partners HealthCare Personalized Medicine (Cambridge, MA, USA) as previously described [19]. Briefly, DNA from the selected individuals were analyzed for candidate causative variants via WES. WES was performed using the Agilent SureSelect Clinical Research Exome capture kit (#G9496A 5190-7344), which captures coding regions (exons) and canonical splice sites of all annotated genes, followed by sequencing on the Illumina HiSeq 2500. Reads were aligned to the GRCh37 reference sequence using the Burrows-Wheeler Aligner (BWA 0.7.17, (http://bio-bwa.sourceforge.net, accessed on 15 April 2021)), and variant calls were made using the Genomic Analysis Tool Kit (GATK v4.0.3.0 (Broad Institute, MA, USA)). The bioinformatics analysis pipeline has been previously described in [19,20,21]. Alignment evaluations were made using SAMtools stats; the overall percentage of the properly paired reads was 98% ± 1, indicating that the proper alignment was achieved (Table S1). Additionally, the evaluations of the called variants were conducted utilizing BCFtools stats (Table S2). Both SAMtools stats and BCFtools stats analyses were made using the Galaxy platform [22]. The average coverage of the variants at >10× was 87.6% ±3.6 (a summary of the exome coverage is shown in Table S3). Noteworthy, large copy number variants (CNVs) were not analyzed in our bioinformatics pipeline. Variant prioritization and filtration were performed using the Illumina BaseSpace variant interpreter tool (https://variantinterpreter.informatics.illumina.com/, accessed on 15 April 2021). We focused our filtration approach on IRD-associated genes, which are reported in the RetNet database (https://sph.uth.edu/retnet/, accessed on 15 April 2021), the OMIM database (https://omim.org, accessed on 15 April 2021) and in the literature (the full gene list has been previously described [19]). 

All the sequence variants were filtered for quality assurance (minimum coverage ≥ 10×, and QD ≥ 4) and location (placed within the exome and/or the flanking intronic regions). Variants with a minor allele frequency of ≤1% in large population databases (ExAC, 1000 genomes project, NHLBI exome sequencing project, gnomAD, the Haplotype Reference Consortium (HRC), KaViar, greater Middle East (GME) variome project and our in-house database of 100 exomes), in addition to loss-of-function variants as well as other variant types that have been previously described in the literature and the Human Gene Mutation Database (HGMD), were prioritized and further analyzed. For the missense variants reported in this study, potential deleterious effects of each variant on the protein structure/function were evaluated using multiple in silico tools, including: Polymorphism Phenotyping v2 (PolyPhen-2), Sorting Intolerant from Tolerant (SIFT), Mutation Taster, Mutation Assessor and Provean.

### 2.3. Sanger Validation and Co-Segregation Analysis

For WES validation, Sanger sequencing was performed for the identified DCVs. Co-segregation analysis was performed for confirmation of the candidate pathogenic variants identified by WES as previously described [19]. Primers sequences are presented in Table S4. Sanger traces were analyzed by Chromas Pro software (Technolysium Ltd., South Brisbane, Australia).

## 3. Results

### 3.1. Patients and Clinical Information

A total of 55 families diagnosed with an IRD (primarily RP and CRD) were recruited for this study; family pedigrees are as shown in Figure S1. Overall, 39 of the participating families (71%) were from consanguineous marriages (Table 1). In 35 families (64%), the inheritance pattern was autosomal-recessive, whereas the probands in 20 families (35%) were from sporadic cases. Among autosomal-recessive IRD families, 11 were previously reported and described [19,23,24]. Age of patients ranged from 4 to 64 years with a mean of 31 years. Best-Corrected Visual Acuity (BCVA) ranged from No Light Perception (NLP) to 0.7, with about 90% (78/87) of patients having a BCVA less than 0.3 in their worst eye. The observed clinical phenotypes following ophthalmic examination are summarized in Table S5. OCT images demonstrated retinal atrophy.

### 3.2. Identification of Potential Pathogenic Variants in the IRD Cohort

**Figure 1:**
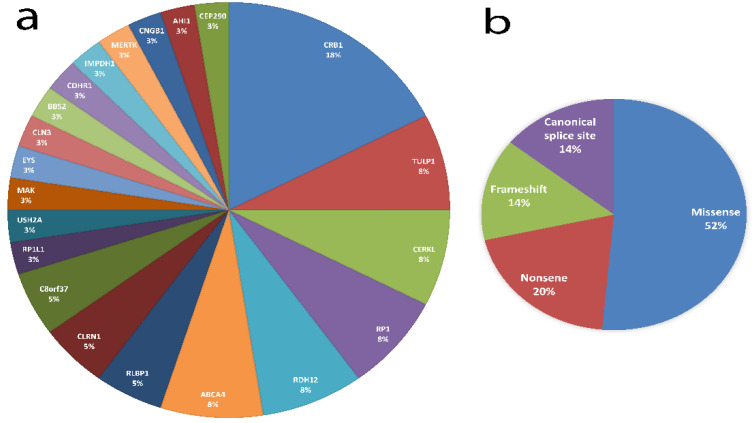
The characteristics of the identified disease-causing variants (DCVs) in our cohort. (**a**) Distribution of candidate DCVs per gene; (**b**) distribution of types within our cohort.

**Figure 2:**
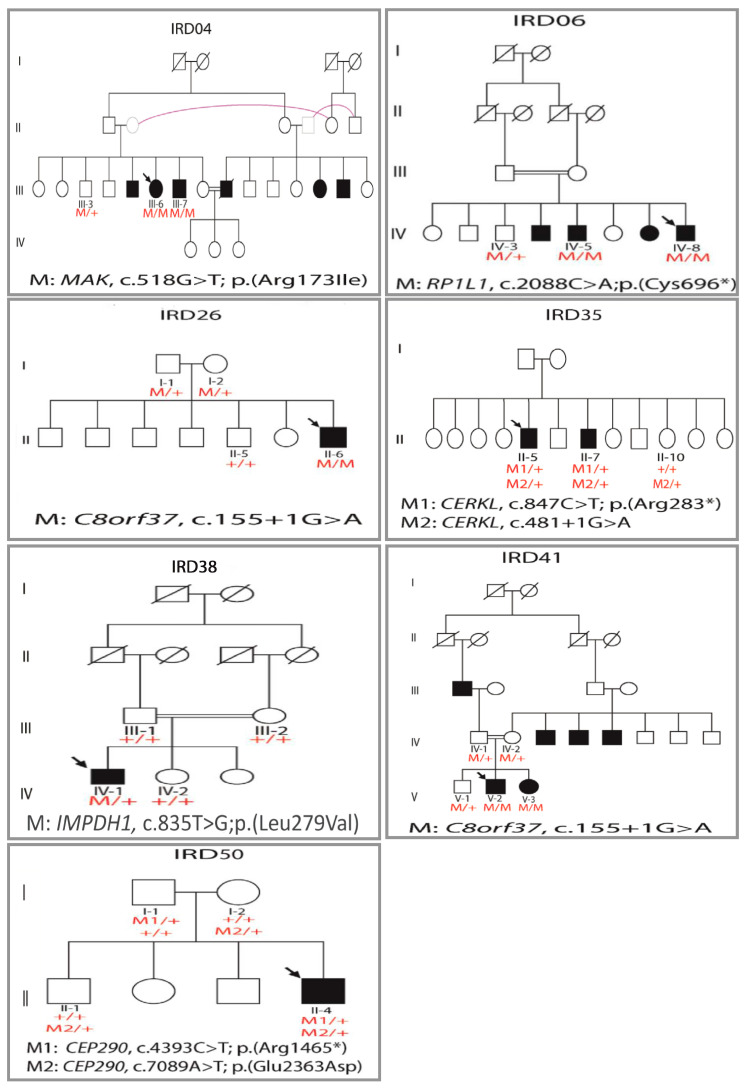
Pedigrees of families with identified novel potential disease-causing variants. Arrows indicate probands. Affected individuals are indicated with filled symbols, unaffected relatives are indicated by open symbols, consanguinity is marked by double lines and pink connected symbols signify the same person. M: mutation; +: wild type allele.

Next, we performed a WES analysis to identify candidate Disease-Causing Variants (DCVs) among the study population. Potential DCVs were found in 39/55 families (71%) (26 autosomal-recessive and 13 sporadic cases) (Table 1 and Table 2). In total, we identified 35 unique potential DCVs in 21 IRD-related genes, of which 24 (68.6%) were likely pathogenic (LP)/pathogenic (P) (Table 2 and Figure 1a). Among the identified variants, 18 (51.4%) are missense variants, 7 (20%) are nonsense variants, 5 (14.3%) are frameshift deletions and 5 (14.3%) are predicted to affect splicing (Figure 1b). Interestingly, of these, 17% (6/35) were novel and included one nonsense, two splicing and three missense variants (Table 2 and Figure 2). The remaining 29 variants have either been previously reported in the literature and/or in the ClinVar database (https://www.ncbi.nlm.nih.gov/clinvar/, accessed on 15 April 2021). 

Potential DCVs were identified in 31 (79.5%) of the 39 consanguineous families, (Table 1). Amongst the 35 families with an autosomal-recessive inheritance pattern, potential DCVs were identified in 26 families with 74.3% detection rate (Table 1). For the sporadic cases, potential DCVs were identified in 65% of the probands (13/20), in which, only one of them (7.7%) harbored a heterozygous variant in a gene implicated in autosomal-dominant form, while the remaining 12 cases (92.3%) harbored homozygous or compound heterozygous variants in genes implicated in autosomal-recessive forms of retinal disease (Table 1). A *de novo* variant was suspected for one of the sporadic cases with an identified heterozygous variant (IRD38)(Figure 2); particularly when no other candidate homozygous variants were detected in the filtered IRD-associated genes. For further assessment, in family IRD38, both parents and one healthy sibling were available for testing, which all of them were clinically unaffected and did not carry the novel variant (*IMPDH1*, het c.835T>G; p.(Leu279Val)) that was detected in the affected proband, confirming it is *de novo*.

Lastly, our genetic investigation was incapable of detecting any candidate DCVs in the relevant IRD-associated genes that could be implicated in the disease phenotype in 16 of the participating families. Our preliminary analysis for 10 of those families filtered candidate variants, however, these variants did not co-segregate with the disease phenotype in the participating family members, which reduces the likelihood of the pathogenicity of these variants (Table S6).

### 3.3. Variant Spectrum in Jordanian Patients with IRD 

The characteristics of the DCVs identified in our cohort are shown in Figure 1. In our variants’ pool, the most frequently mutated gene was the *CRB1* (18%, 7/39 families, representing 4 missense variants) (Table 2 and Figure 1a). Interestingly, one of the *CRB1* variants was present in three unrelated families; c.1733T > A; p.(Val578Glu) (Table 2). Additionally, 19 loss-of function variants were identified in our cohort in *TULP1, CERKL, CLRN1, RP1, RLBP1, C8orf37 ABCA4, USH2A, EYS, CDHR1, RP1L1, RDH12* and *CEP290*, of which three were novel: a splice-donor variant in *C8orf37*, a splice-donor variant in *CERKL* and a nonsense variant in *RP1L1*. The splice donor variant in *C8orf37* (c.155 + 1G > A) was identified in two unrelated families (IRD26 and IRD41). This variant is predicted to affect splicing, potentially leading to abnormal or absent proteins.

### 3.4. Phenotypic and Genotypic Information

**Figure 3:**
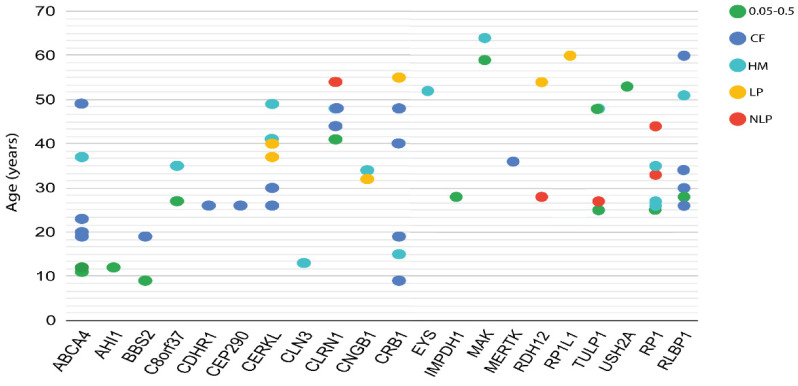
Correlation between visual acuity, age at exam and genes in which potential DCVs were identified. The phenotypic severity of visual acuity is classified into five groups, which are plotted in different colors. CF: counting fingers, HM: hand motion, LP: light perception, NLP: no light perception. Genes are plotted on the X-axis, while the Y-axis represents the patients’ ages at exam.

To investigate the relationship between variants identified in IRD genes and the clinical features observed in our cohort, we performed genotype–phenotype analyses of all IRD patients in whom a potential DCV was detected. Clinical data for all patients, including the most recent BCVA and age at examination, are shown in a scatterplot across the 21 IRD-associated genes harboring the identified variants (Figure 3). We classified the visual acuity into five groups depending on the phenotypic severity, each severity is represented by different color code (Figure 3). In this cohort, although the BCVA on average was less than 0.3 in most patients (Figure 3), we found no obvious correlation between BCVA severity, age at examination and genotype in any specific gene.

### 3.5. Investigating the Less Commonly Studied Genotype–Phenotype Correlations

Several IRD genes have been commonly associated with a specific retinal phenotype in the literature [3]. We performed detailed clinical phenotyping on our patients and found that, in some cases, certain genes are responsible for an IRD phenotype that is less typically associated with that gene. These less-established genotype–phenotype correlations for several genes with DCVs identified in our cohort are described below.

**Figure 4:**
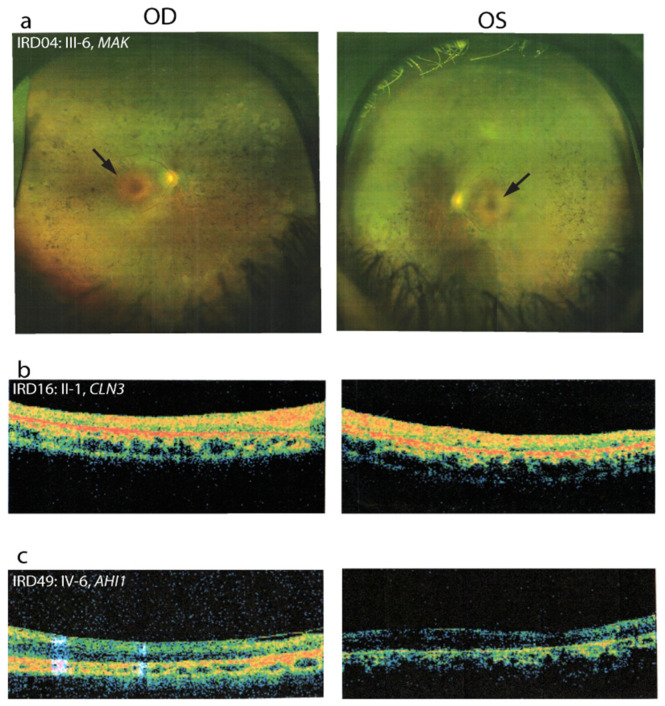
Optical Coherence Tomography (OCT) or fundus photography of both eyes for identified families with interesting genotype-phenotype correlations: (**a**) fundus photography for patient IRD04: III-6, arrow points to bull’s eye maculopathy; (**b**) patient IRD16: II-1 OCT showing loss of foveal reflex with generalized macular thinning, no cystoid macular edema or vitreoretinal interface abnormalities or epiretinal membrane; (**c**) patient IRD049: IV-6 OCT showing severe thinning and atrophy.

*MAK*. Variants in *MAK* have been mainly associated with RP (OMIM: 614181) and less commonly implicated with CRD [15]. In our cohort, the family IRD04 carried a novel missense variant (c.518G > T; p.Arg173Ile) in *MAK*. This variant is predicted in silico to have a damaging effect and it is conserved across different species (Table 2). A co-segregation analysis confirmed that the variant segregated with the disease phenotype in 2 affected siblings (Figure 2). Interestingly, the clinical diagnosis for this family was more consistent with CRD (rather than RP), in which bull’s eye maculopathy was also evident (Figure 4a). These results are suggestive of an association between this *MAK* variant with bull’s eye maculopathy and CRD. 

*CLN3*. A sporadic case of an early adolescent RP female patient in family IRD16 was found to have a previously described missense variant (c.1000C > T; p.(Arg334Cys)) in *CLN3*. This variant is predicted in silico to have a damaging effect and it is conserved across different species (Table 2). On examination, BCVA was hand motion (HM) for her worst eye. Fundoscopy showed minimal bone spicule pigmentation, while OCT images showed loss of foveal reflex with generalized macular thinning; no cystoid macular edema or vitreoretinal interface abnormalities or epiretinal membrane were identified (Figure 4b). *CLN3* is classically associated with juvenile neuronal ceroid lipofuscinosis (JNCL) 3 disease (OMIM: 607042), which primarily affects the nervous system and is characterized by vision impairment, cognitive disability, movement problems, speech difficulties and seizures that develop during childhood [69]. The involvement of *CLN3* in nonsyndromic RP has been emerging recently [70,71,72,73,74,75]. The identified variant was previously reported in a patient with clinical features meeting the diagnostic criteria for JNCL [57]. Interestingly, the proband from family IRD16 showed only a typical RP phenotype without systemic manifestation, at least for this age as an adolescent.

*AHI1*. *AHI1* is originally known to cause Joubert syndrome 3 (OMIM: 608894); a congenital multi-organ disease involving the retina, kidneys, bones and liver [76]. Nonsyndromic RP association has been recently, and less frequently, associated with *AHI* [75]. A missense variant (c.2335G > A; p.(Asp779Asn)) in the *AHI1* gene was identified in the homozygous state in an early adolescent male patient (sporadic case, IRD49) who had isolated RP. Co-segregation analysis revealed that both parents were heterozygous carriers for the variant and his unaffected sibling is homozygous for the wild-type allele. The patient’s BCVA was 0.2 and 0.05 for his right and left eye, respectively. The classic RP triad, i.e., attenuated blood vessels, bony spicules and macular degeneration, were observed upon fundus examination. OCT showed severe thinning and atrophy (Figure 4c). The variant has only been reported by another clinical laboratory in ClinVar and has been interpreted in the context of Joubert Syndrome, whereas the proband from family IRD49 had nonsyndromic RP (Table 2).

## 4. Discussion

Molecular diagnosis of IRDs remains challenging given the high genetic heterogeneity of this group of disorders. In this study, we described the variant spectrum detected in a total of 55 Jordanian families with IRDs, which, to our knowledge, is the largest Jordanian IRD cohort studied to date. Potential pathogenic variants were detected in 39 (out of 55) of the analyzed families (71% detection rate). The worldwide reported detection rates in other IRD cohorts ranged between 41% and 76% and as follows: Saudi-Arabia (54%) [77], Israel (49%, 56%) [78,79], China (41.4%) [80], Switzerland (64%) [81], England (56%) [40], Ireland (68%) [82], Germany (70.8%) [83] and the USA (76%) [32]. These cohort studies, as well as our own, have demonstrated that NGS is a reliable approach in detecting the underlying molecular etiology of IRDs. The variation of detection rates could be explained by several factors including the utilized genetic platforms, panel design, inclusion criteria, the genetic heterogeneity of the investigated phenotype and the number of participating family members of the affected probands. The detection rate is also dependent on the level of consanguinity between the study subjects. We were able to identify the DCVs in 79.5% of the consanguineous families. Similarly, other studies also investigated the genetic basis of IRDs in consanguineous families, for instance, in Pakistani [84] and Iranian [85] in-breeding kindreds, they were able to identify the genetic etiology in around 72% and 90% of the cases, respectively. 

Among the 21 IRD-associated genes found to contain potential pathogenic variants in our cohort, variants were most prevalent in *CRB1, TULP1, CERKL, RDH12, ABCA4* and *RP1*, which collectively represent 40% of variants identified in the entire cohort and more than 56% of cases where a potential DCV was identified. These findings suggest that these genes should be considered upon designing gene panel-based tests for the diagnosis of IRD in Jordan, which might serve as a more economical alternative to utilizing WES. Interestingly, variants in *CRB1* account for the majority of autosomal-recessive early-onset retinal degeneration cases in Israeli and Palestinian populations, as well as LCA cases in a Japanese population [25,86]. *CERKL* was the most common gene implicated in a Tunisian population with retinal dystrophy [87], while *RDH12* was also common in the Israeli population with inherited retinopathies [78].

Our analysis has also identified novel variants (17%) that are not previously reported, suggesting their uniqueness to Jordanian, or potentially, Middle Eastern populations. Thus, there may be several yet-to-be-identified clinically relevant variants in the context of IRD in this population. In an attempt to describe less commonly reported genotype-phenotype correlations, we assessed the relationships between the severity of visual acuity as well as age at examination, and specific genotypes across various IRD-associated genes. However, no clear correlations were observed (Figure 3). These results reinforce the fact that IRDs show clinical and genetic heterogeneity. Nevertheless, an examination of a larger cohort and the identification of more variants causing IRD are warranted to further elucidate any genetic associations in our findings.

Interestingly, in three families (IRD04, IRD16 and IRD49), potential DCVs were detected in genes for which an association with the observed IRD phenotype in the family was different than the commonly reported phenotype. Our results suggest a candidate variant in *MAK* to be implicated in CRD. To date, all 23 pathogenic variants that have been reported in *MAK* gene have been mainly associated with RP, and are rarely implicated with CRD. Interestingly, one patient from family IRD04 showed symptoms consistent with CRD, where bull’s eye maculopathy was present. In families IRD16 and IRD49, a potential DCV was detected in *CLN3* and *AHI1*, respectively. *CLN3* and *AHI1* typically cause JNCL 3 and Joubert syndrome 3, respectively; however, probands of families IRD16 and IRD49 showed typical RP phenotypes, without any additional syndromic manifestations of either disease, at least for their current age. Although the identified variants in CLN3 and AHI1 genes were previously reported, in this study, an association with less commonly reported phenotypes can be inferred. In 16 of our IRD families, we were unable to identify a variant that could potentially explain the observed phenotypes. This highlights the genetic complexity of retinal degenerative diseases and the limitations of WES, in that certain variants in current disease-associated genes can be missed by WES (e.g., deep intronic changes that could affect mRNA splicing, 5′ and 3′ UTR changes affecting mRNA production and stability, structural variants (SV) and CNVs, which can include large deletions/insertions of one or more full exons) or cannot be clinically interpreted due to lack of case-level and functional data. Lastly, it cannot be excluded that additional retinopathy-associated genes remain to be identified. 

## 5. Conclusions

In summary, this is the largest comprehensive genomic study of IRD patients from the Jordanian population known to date. In this study, we showed that WES is very useful in identifying disease-causing variants in a Jordanian IRD cohort that has not undergone prior genetic analysis. Our NGS-based diagnostic approach successfully identified a putative variant of clinical significance in 39/55 IRD families. Six of these variants were novel, without any reported association to disease. The results from this study expand the variant spectrum of the Jordanian population with IRD and contribute to better understanding of molecular mechanisms of the disease. Finally, this approach provides an avenue to facilitate the clinical diagnosis and personalized treatment of patients with IRD.

## Figures and Tables

**Table 1:** Distribution and detection rates in patients with inherited retinal dystrophy.

Families	AR	Sporadic	Total	Consanguineous
Solved (*n*)	26	13	39	31
Unsolved (*n*)	9	7	16	8
Total, *n* (%)	35 (64%)	20 (36%)	55 (100%)	39 (71%)
Detection rate of potential DCVs (%)	74.3%	65%	71%	79.5%

**Table 2:** Candidate disease variants identified in the Jordanian inherited retinal dystrophies cohort.

Family ID	Gene	Variant Coordinate hg19	HGVS Variant Nomenclature	dbSNP ID	gnomAD v3.1.1 Frequency			Zygo.	Segregation	ClinVar *	In silico Predictions SIFT, PP, MT	ACMG Classification	References
					Highest	SAS	ME $						
IRD03	*CRB1*	Chr1:197404300	NM_201253.2:c.3307G > A; p.(Gly1103Arg)	rs62636275	2.1 × 10^−4^	2.1 × 10^−4^	NA	Hom	Not done	P	D, D, A	LP	[25,26,27,28,29]
IRD47	*CRB1*	Chr1:197404300	NM_201253.2:c.3307G > A; p.(Gly1103Arg)	rs62636275	2.1 × 10^−4^	2.1 × 10^−4^	NA	Hom	Not done	P	D, D, A	LP	[25,26,27,28,29]
IRD14	*CRB1*	Chr1:197390691	NM_201253.2:c.1733T > A; p.(Val578Glu)	rs1266363944	NA	NA	NA	Hom	Not done	LP	D, D, DC	VUS	[25,30]
IRD19	*CRB1*	Chr1:197390691	NM_201253.2:c.1733T > A; p.(Val578Glu)	rs1266363944	NA	NA	NA	Hom	Not done	LP	D, D, DC	VUS	[25,30]
IRD28	*CRB1*	Chr1:197390691	NM_201253.2:c.1733T > A; p.(Val578Glu)	rs1266363944	NA	NA	NA	Hom	Yes	LP	D, D, DC	VUS	[25,30]
IRD33	*CRB1*	Chr1:197396763	NM_201253.2:c.2308G > A; p.(Gly770Ser)	rs767648174	6.6 × 10^−5^	NA	NA	Hom	Not done		D, D, DC	LP	[31,32,33]
IRD39	*CRB1*	Chr1:197390802	NM_201253.2:c.1844G > T; p.(Gly615Val)		1.5 × 10^−5^	NA	NA	Hom	Yes		D, D, DC	LP	[25]
IRD09	*TULP1*	Chr6:35473549	NM_003322.3:c.1081C > T; p.(Arg361 *)		2.4 × 10^−5^	NA	NA	Hom	Yes			P	[34,35,36]
IRD12	*TULP1*	Chr6:35467755	NM_003322.3: c.1495 + 2dupT	rs1581735836	NA	NA	NA	Hom	Yes			P	[37]
IRD31	*TULP1*	Chr6:35473543	NM_003322.3:c.1087G > A; p.(Gly363Arg)		4.8 × 10^−4^	NA	NA	Hom	Not done		D, D, DC	VUS	[38]
IRD11	*CERKL*	Chr2:182468594	NM_001030311.2: c.450_451delAT; p.(Ile150Metfs * 3)		NA	NA	NA	Hom	Yes			P	[19]
IRD18	*CERKL*	Chr2:182413318	NM_001030311.2: c.1164_1165delTG; p.(Cys388 *)	rs776727320	1.1 × 10^−3^	NA	NA	Hom	Yes			P	[19,39]
IRD35	*CERKL*	Chr2:182423344	NM_001030311.2: c.847C > T; p.(Arg283 *)	rs121909398	9.6 × 10^−4^	NA	NA	Com. het	Yes	P		P	[32,40,41,42,43,44,45,46,47,48,49,50,51,52]
**IRD35**	***CERKL*** ****	**Chr2:182468563**	**NM_001030311.2: c.481 + 1G > A**		**NA**	**NA**	**NA**	**Com.het**	**Yes**			**P**	**Novel**
IRD02	*CLRN1*	Chr3:150659368	NM_001195794.1: c.433 + 1G > A	rs201205811	NA	NA	NA	Hom	Yes			P	[23]
IRD36	*CLRN1*	Chr3:150659479	NM_001195794.1:c.323T > C; p.(Leu108Pro)		4.6 × 10^−4^	NA	NA	Hom	Yes		D, D, DC	VUS	[23]
IRD05	*RP1*	Chr8:55537568	NM_006269.1: c.1126C > T; p.(Arg376*)	rs760689800	NA	NA	NA	Hom	Yes			P	[24]
IRD08	*RP1*	Chr8:55534133	NM_006269.1: c.607G > A; p.(Gly203Arg)	rs786205589	NA	NA	NA	Hom	Yes	LP	D, D, DC	LP	[24]
IRD22	*RP1*	Chr8:55534133	NM_006269.1: c.607G > A; p.(Gly203Arg)	rs786205589	NA	NA	NA	Hom	Yes	LP	D, D, DC	LP	[24]
IRD10	*RLBP1*	Chr15:89761858	NM_000326.4: c.79delA; p.(Thr27Profs * 26)	rs1567124404	NA	NA	NA	Hom	Yes	P		P	[24]
IRD17	*RLBP1*	Chr15:89758418	NM_000326.4: c.398delC; p.(Pro133Glnfs * 126)	NA	NA	NA	NA	Hom	Yes			P	[24]
**IRD26**	***C8orf37*** ****	**Chr8:96281262**	**NM_177965.3: c.155 + 1G > A**		**6.5 × 10** ^**−5**^	**NA**	**NA**	**Hom**	**Yes**			**P**	**Novel**
**IRD41**	***C8orf37*** ****	**Chr8:96281262**	**NM_177965.3: c.155 + 1G > A**		**6.5 × 10** ^**−5**^	**NA**	**NA**	**Hom**	**Yes**			**P**	**Novel**
IRD02	*ABCA4*	Chr1:94480098	NM_000350.2: c.5460 + 1G > A	rs61753030	2.4 × 10^−5^	NA	NA	Hom	Yes			P	[23]
IRD24	*ABCA4*	Chr1:94528780	NM_000350.2: c.1648G > A; p.(Gly550Arg)	rs61748558	1.4 × 10^−5^	NA	NA	Hom	Yes	LP	D, D, DC	LP	[23]
IRD48	*ABCA4*	Chr1:94480098	NM_000350.2: c.5460 + 1G > A	rs61753030	2.4 × 10^−5^	NA	NA	Hom	Yes			P	[23]
IRD37	*USH2A*	Chr1:216019303	NM_206933.2: c.8917_8918del; p.(Leu2973Lysfs * 79)		NA	NA	NA	Hom	Not done			P	[53]
**IRD04**	***MAK*** ****	**Chr6:10804098**	**NM_001242957.1: c.518G > T; p.(Arg173Ile)**		**NA**	**NA**	**NA**	**Hom**	**Yes**		**D, D, DC**	**VUS**	**Novel**
IRD07	*EYS*	Chr6:65655759	NM_001142800.1: c.2308C > T; p.(Gln770 *)		NA	NA	NA	Hom	Not done			P	[54,55]
IRD16	*CLN3*	Chr16:28493482	NM_001042432.1: c.1000C > T; p.(Arg334Cys)	rs386833694	NA	NA	NA	Hom	Not done	LP	D, D, DC	VUS	[56,57]
IRD20	*BBS2*	Chr16:56536365	NM_031885.3:c.944G > A; p.(Arg315Gln)	rs544773389	NA	NA	NA	Hom	Not done	VUS	D, D, DC	VUS	[58,59]
IRD27	*CDHR1*	Chr10:85957581	NM_033100.3:c.338delG; p.(Gly113Alafs * 2)	rs747425652	NA	NA	NA	Hom	Yes			P	[60]
**IRD38**	***IMPDH1*** ****	**Chr7:128040188**	**NM_000883.3:c.835T > G; p.(Leu279Val)**		**NA**	**NA**	**NA**	**Het**	**Yes**		**D, D, DC**	**LP**	**Novel**
IRD46	*MERTK*	Chr2:112779847	NM_006343.2:c.2362G > A; p.(Val788Met)	rs769691218	6.5 × 10^−5^	NA	NA	Hom	Not done		D, D, DC	VUS	ClinVar
IRD56	*CNGB1*	Chr16:57937858	NM_001297.4:c.2662G > A; p.(Ala888Thr)	rs368328328	8.3 x10^−4^	8.3 × 10^−4^	NA	Hom	Yes		D, D, DC	VUS	ClinVar
**IRD06**	***RP1L1*** ****	**Chr8:10469520**	NM_178857.5:c.2088C > A; p.(Cys696 *)		**NA**	**NA**	**NA**	**Hom**	**Yes**			**P**	**Novel**
IRD21	*RDH12*	Chr14:68192803	NM_152443.2:c.379G > T; p.(Gly127 *)	rs104894474	NA	NA	NA	Hom	Not done	P		P	[47,61,62]
IRD55	*RDH12*	Chr14:68196070	NM_152443.2:c.821T > C; p.(Leu274Pro)		NA	NA	NA	Hom	Yes		D, D, DC	VUS	[63,64]
IRD25	*RDH12*	Chr14:68196070	NM_152443.2:c.821T > C; p.(Leu274Pro)		NA	NA	NA	Hom	Yes		D, D, DC	VUS	[63,64]
IRD49	*AHI1*	Chr6:135752384	NM_017651.4:c.2335G > A; p.(Asp779Asn)		3.2 × 10^−3^	NA	3.2 × 10^−3^	Hom	Yes	VUS	T, D, N	VUS	ClinVar
IRD50	*CEP290*	Chr12:88479860	NM_025114.3:c.4393C > T; p.(Arg1465 *)	rs539400286	2.1 × 10^−4^	2.1 × 10^−4^	NA	Com. het	Yes	P		P	[46,47,65,66,67,68]
**IRD50**	***CEP290***	**Chr12:88447469**	**NM_025114.3:c.7089A > T; p.(Glu2363Asp)**		**NA**	**NA**	**NA**	**Com. het**	**Yes**		**T, B, N**	**VUS**	**Novel**

* At least one star status. $ All the identified DCVs were not reported in the GME variome database. PP: PolyPhen, MT: MutationTaster, DC: Disease Causing, A: Disease causing Automatic, D: damaging, N: neutral, B: benign, T: tolerated, NA: not available, VUS: variant of unknown significance; P: pathogenic; LP: likely pathogenic, Het: heterozygous, Hom: homozygous, Zygo.: zygosity; Com. het: compound heterozygous; SAS: South Asia, ME: Middle East.

## Data Availability

The data that support the findings of this study are available in the supplementary material of this article. Any additional required data that support the findings of this study are available from the corresponding author upon reasonable request.

## Supplementary Materials

The following are available online at https://www.mdpi.com/article/10.3390/genes12040593/s1, Figure S1: Pedigrees for all the participating families with inherited retinal dystrophies, Table S1: Alignment evaluation summary for each of the participating families using SAMtools stats, Table S2: Variant evaluation summary for each of the participating families using BCFtools stats, Table S3: Exome coverage summary for each of the participating families, Table S4: Primers used for the validation and segregation analysis of the identified variants, Table S5: Clinical features of an inherited retinal dystrophy (IRD) cohort, Table S6: Candidate variants with low evidence of pathogenicity identified in a Jordanian IRD cohort.
