# Supplementary material for: Unique Variant Spectrum in a Jordanian Cohort with Inherited Retinal Dystrophies
Source: Genes (Basel). 2021 Apr 19;12(4):593. doi: 10.3390/genes12040593 (PMC8074154; doi:10.3390/genes12040593)
Supplement: Supplementary file 1 [file genes-12-00593-s001.zip › Table S4,S5,S6.pdf]

Supplementary Material

Unique Variant spectrum in a Jordanian cohort with inherited retinal dystrophies

Bilal Azab[1, 2†*], Zain Dardas[2, 3†], Dunia Aburizeg[2], Muawyah Al-Bdour[4], Mohammed Abu-Ameerh[4], Tareq Saleh[5], Raghda Barham[6], Ranad Maswadi[7], Nidaa A Ababneh[6], Mohammad Alsalem[8], Hana Zouk[9, 10], Sami Amr[11], Abdalla Awidi[6]**

[1] Department of Human and Molecular Genetics, School of Medicine, Virginia Commonwealth University, Richmond, VA 23298-0565, USA
[2] Department of Pathology and Microbiology and Forensic Medicine, School of Medicine, The University of Jordan, Amman 11942, Jordan; zain.dardas@bcm.edu (Z.D.); dunia.aburizeg@gmail.com (D.A.)
[3] Department of Molecular and Human Genetics, Baylor College of Medicine, Houston, TX 77030, USA
[4] Department of Ophthalmology, Jordan University Hospital, The University of Jordan, Amman 11942, Jordan; bdourjo@yahoo.com (M.A.-B.); mohammd_73@yahoo.com (M.A.-A.)
[5] Department of Basic Medical Sciences, Faculty of Medicine, The Hashemite University, Zarqa 13115, Jordan; tareq@hu.edu.jo
[6] Cell Therapy Center, The University of Jordan, Amman 11942, Jordan; raghda.barham@gmail.com (R.B.); nidaaanwar@gmail.com (N.A.A.)
[7] Department of Ophthalmology, St Thomas' Hospital, London SE1 7EH, UK; Ranad.maswadi@gstt.nhs.uk
[8] Department of Anatomy and Histology, School of Medicine, The University of Jordan, Amman 11942, Jordan; dralsalem@gmail.com
[9] Laboratory for Molecular Medicine, Partners HealthCare Personalized Medicine, Cambridge, MA 02139, USA; hzouk@bwh.harvard.edu
[10] Department of Pathology, Massachusetts General Hospital/Harvard Medical School, Boston, MA 02114, USA
[11] Department of Pathology, Brigham and Women's Hospital, Harvard Medical School, Boston, MA 02115, USA; samr@bwh.harvard.edu
† These authors contributed equally to this work.
* Correspondence: azabbm@mymail.vcu.edu (B.A.); abdalla.awidi@gmail.com (A.A.); Tel.: +96-279-840-2240 (B.A.); Tel.: +96-279-527-7455 (A.A.)

Table S4: Primers used for the validation and segregation analysis of the identified variants.

Gene	Forward primer	Reverse primer	Amplicon size	Tm.
TULP1	ACTTATGCCCTGCTCCAAGT	GGCATGGATCGAGGCATGTA	600	60
C8orf37	AGTCTGCGCTGAGTGAAGAG	CCGAACTCTGTCCCTGCATA	569	60
CRB1	ACATGCATCCCTCACTTCCA	ACAAGGTTGGCTTTCACACC	681	60
IMPDH1	GAGCATCGCATAGGACTAGTT	GCAGAAGTTTGAACAGGGCT	686	60
AHI1	CAGTGCAAATGGGGTATAAAA	ACACATGTACTGAGAGGCTC	638	60
CEP290	TCCTTTCATATTCAGTGTACCGA	TGAAATACTAAATGCGGCACAAA	613	60
RDH12	TGGTACCTGCTGAATCCTGG	GCATAACCAACAGCGACAGT	610	60
MAK	AGCAACTTAGGGACAAGAGTACT	ATTTGTCTCCAGCCCCACTC	414	60
RP1L1	TTTGAGCAGGAGTCGGATGT	CTTCCACCTGCACTTCATCC	475	60
TULP1	ATGGAGGAGAGAGTGACCCT	ATGAAGATGTCCAGGCCACA	441	60
RDH12	TGGTACCTGCTGAATCCTGG	GCATAACCAACAGCGACAGT	610	60
CDHR1	CTGTCACCACCACTTACCCT	GGGCAAGTTTGAGGAGATGC	472	60
CRB1	ACATGCATCCCTCACTTCCA	ACAAGGTTGGCTTTCACACC	681	60
CNGB1	CAGAGACACAGAGGACGAGG	GGCAGAACTTCCCGTCAGTA	445	60
CERKL	GCCATTGGGGAGTTATTCCT	TCTGTGTTGGTGGAGATGGA	456	60
ERCC6	TACCTGTTGCCTGATGTCCC	GTTAGTGCTGCCATGAGACG	484	60
PRSS56	CCGAATGAGCTTCTGTGGAC	CCCTTTGAAAGAGCAGGCAG	454	60
KCNJ13	CAGGGAGAAGGAGAATGCAG	AGCATTCCCAAGAAAGCAGA	455	60
RGR	TCTTCTGTGTGACCTTGGCT	CCCAAGGTTGCGGTAATGG	699	60
PITPNM3	CTCACTGCAAACTCCACCTC	CTCTAGCCCAAGTCTGACCC	716	60
HMCN1	GGCACTTGATGAGAACTTCCC	GGAAGACTGAAGGTGAGGCA	707	60
OPN1SW	TTTCCCCCAGACTCCTCTTT	AGCTTTCATGGGCACTGTCT	323	60
ROM1	TGACACCTCTGCATTCCCTT	GTAACCAGTCTCTCAGCCACT	750	60
CAPN5	CACAGCCTCCTCAGTCTGAC	GCCCAGTAGATGCCCAGAG	754	60
COL2A1	ACCCAAGCTGAGGAATCCC	TGAGTTGGGCAGAAGAGGAG	779	60
COL9A1	AGCTGAATGATCGTTTACCAGA	TGCTACCTCCCTCCCCGT	769	60
PAX2	ACATCCCATGCCTCTCAACA	ATTCTGGGCAACTTGGGAGA	764	60
ADGRV1	AAAATTTGCTTATGTTGCCCA	CCAGTAGAGAATGGCAAGC	589	60

Table S5: Clinical features of inherited retinal dystrophy (IRD) cohort.

Family ID	Consanguinity	Implicated gene GeneBank Accession No. [DCVs]	Patient ID	Age (yrs)		BCVA		CORNEA	LENS		IOP		Fundus exam
				At exam	Onset	OD	OS		OD	OS	OD	OS	
IRD01	No	-	010210	42	5	0.4	0.4	Clear	No L.O	No L.O	Soft	Soft	Scattered bony spicules
			010208	51	10	HM	HM	Clear	L.O	L.O	Soft	Soft	Bony spicules, Pale disk
IRD02	Yes	*CLRN1* NM_001195794.1 [c.433+1G>A]	020416	48	5	HM	CF closely	Clear	NS+1	PSCC+2	14	12	Bony spicules, Pale disk
			020417	54	7	NLP	0.4	Clear	Clear	PCIOL	19	14	RP triad
			020418	44	6	CF at 1 m	CF at 1.5m	Clear	NS+1+PSCC	NS+1+ PSCC	14	14	RP triad
			020419	48	7	CF at 2 m	CF at 2 m	Clear	PSCC+2	PSCC+2	Soft	Soft	RP triad
		ABCA4 NM_000350.2 [c.5460+1G>A]	020407	49	10	CF at 1.25 m	0.05	Clear	No L.O	No L.O	Soft	Soft	RP triad
			020408	37	10	HM	HM	Clear	Clear	Clear	Soft	Soft	RP triad
			020506	11	9	0.1	0.1	Clear	No L.O	No L.O	Soft	Soft	Attenuated arterioles and RPE changes

Table S5 Abbreviations: DCVs: Disease-causing variants, BCVA: Best Corrected Visual Acuity, IOP: Intraocular Pressure, L.O: Lenticular opacity, HM: Hand Motion, CF: Counting Fingers, NS: Nuclear Sclerosis, PSCC: Posterior subcapsular Cataract, PCIOL: Posterior Chamber Intraocular Lens, IOL: Intraocular Lens, LP: Light Perception, NLP: No Light Perception, PCO: Posterior capsule opacification, PVD: Posterior vitreous detachment, ASCC: Anterior subcapsular cataract, PPA: Peripapillary atrophy, NA: Not Available, CSM: central, steady, maintained. RP triad: Bony spicules, attenuated blood vessels, and pale optic disk.

			020507	12	6	0.1	0.1	Clear	Clear	Clear	Soft	Soft	Bony spicules, RPE changes
IRD03	No	*CRB1* NM_201253.2 [c.3307G>A]	030203	15	2	HM	CF closely	Clear	Clear	Clear	Soft	Soft	RP triad
			030202	9	2	CF closely	CF closely	Clear	Clear	Clear	Soft	Soft	RP triad
IRD04	No	*MAK* NM_001242957.1 **[c.518G>T]**	040306	59	33	0.5	0.1	Clear	PCIOL	PCIOL	Soft	Soft	Bony spicules, Pale disk
			040307	64	30	HM	0.4	Clear	Sulcus IOL	Sulcus IOL	15	15	RP triad
IRD05	Yes	*RP1* NM_006269.1 [c.1126C>T]	050406	33	6	LP	NLP	Clear	PSCC	Aphakia	12	12	RP triad
			050407	44	6	LP	LP	Clear	Clear	Clear	Soft	Soft	RP triad
			050408	25	6	HM	HM	Clear	No L.O	No L.O	Soft	Soft	RP triad
IRD06	Yes	*RP1L1* NM_178857.5 **[c.2088C>A]**	060408	60	NA	LP	LP	Clear	NS+1	PSCC+2	12	12	Bony spicules, Attenuated blood vessels, and extensive asteroid hyalosis
IRD07	Yes	*EYS* NM_001142800.1 [c.2308C>T]	070406	52	5	HM	HM	Clear	PSCC+3	PSCC+3	12	12	RP triad
IRD08	Yes	*RP1* NM_006269.1 [c.607G>A]	080405	25	6	0.4	0.3	Clear	No L.O	No L.O	13	13	RP triad
			080406	35	5	HM	HM	Clear	PSCC+2	PSCC+2	Soft	Soft	RP triad
IRD09	Yes	*TULP1* NM_003322.3 [c.1081C>T]	090413	48	6	HM	LP	Clear	L.O	L.O	Soft	Soft	RP triad
IRD10	Yes	*RLBP1* NM_000326.4 [c.79delA]	100413	34	1	CF at 2.5 m	CF at 2.5 m	Clear	Blue dots	Blue dots	Soft	Soft	sincRP pigmento
			100410	30	1	CF at 1m	CF closely	Clear	Clear	Clear	Soft	Soft	sincRP pigmento

			100411	28	1	0.05	0.05	Clear	Clear	Clear	Soft	Soft	sincRP pigmento
			100412	26	1	CF closely	CF at 2m	Clear	Clear	Clear	Soft	Soft	sincRP pigmento
IRD11	Yes	*CERKL* NM_001030311.2 [c.450_451delAT]	110406	49	12	HM	HM	Clear	PSCC	PSCC	Soft	Soft	RP triad
IRD12	Yes	*TULP1* NM_003322.3 [c.1495+2dupT]	120405	25	6	0.05	0.05	Clear	No L.O	No L.O	Soft	Soft	RP triad
			120406	27	6	CF at 0.5 m	CF at 0.2 m	Clear	No L.O	No L.O	12	12	RP triad, macular hyperpigme-ntation
IRD13	Yes	-	130404	18	6	CF at 1.5 m	CF at 2.0 m	Clear	No L.O	No L.O	Soft	Soft	RP triad
IRD14	Yes	*CRB1* NM_201253.2 [c.1733T>A]	140403	15	NA	CF at 0.5 m	CF at 1.25m	Clear	No L.O	No L.O	10	8	RP triad
IRD15	Yes	-	150510	37	10	LP	HM	Clear	PSCC	PSCC	Soft	Soft	RP triad
IRD16	No	*CLN3* NM_001042432.1 [c.1000C>T]	160201	13	6	HM	CF closely	Clear	Clear	Clear	Soft	Soft	Minimal bony spicules, ERM, and peripheral retinal flecks
IRD17	Yes	*RLBP1* NM_000326.4 [c.398delC]	170408	60	2	CF closely	CF closely	Clear	L.O	L.O	Soft	Soft	Yellow-white dots at the level of the RPE and concentrated in the retinal midperiphery.
IRD18	Yes	*CERKL* NM_001030311.2 [c.1164_1165delTG]	180515	41	17	HM	HM	Clear	No L.O	No L.O	NA	NA	RP triad with PVD
			180517	30	17	CF closely	HM	Clear	No L.O	No L.O	13	13	RP triad with PVD
			180526	26	28	CF at 2.3 m	CF at 1.5 m	Clear	No L.O	No L.O	NA	NA	RP triad with PVD
			180530	40	15	LP	LP	Clear	No L.O	No L.O	NA	NA	RP triad
IRD19	Yes	*CRB1* NM_201253.2 [c.1733T>A]	190403	40	NA	CF closely	NLP	Clear	L.O	Mature cataract	12	15	Pale disk, Attenuated blood vessels
IRD20	Yes	*BBS2* NM_031885.3 [c.944G>A]	200405	19	NA	CF closely	CF closely	Clear	Clear	Clear	Soft	Soft	Pigmentary changes, RPE atrophy,

													and bony spicules
			200406	9	NA	0.2	0.2	Clear	Clear	Clear	Soft	Soft	RP triad
IRD21	Yes	*RDH12*<br>NM_152443.2<br>[c.379G>T]	210406	28	NA	NLP	HM	Clear	L.O	Dense PCO	Soft	Soft	Severe bony spicules involving the macula, pale disk, attenuated blood vessels
IRD22	Yes	*RP1*<br>NM_006269.1<br>[c.607G>A]	220404	26	5	HM	HM	Clear	PSCC	PSCC	8	8	RP triad
IRD23	Yes	-	230403	21	13	0.3	0.2	Clear	L.O	L.O	12	10	Small tilted disk, attenuated blood vessels, bony spicules
IRD24	Yes	*ABCA4*<br>NM_000350.2<br>[c.1648G>A]	240405	20	11	CF at 1m	0.05	Clear	No L.O	Congenital cataract	10	10	RP triad
IRD25	Yes	*RDH12*<br>NM_152443.2<br>[c.821T>C]	250410	54	28	LP	LP	Arcus senilis	PSCC	PSCC	10	10	Severe bony spicules involving the macula
IRD26	No	*C8orf37*<br>**NM_177965.3**<br>[c.155+1G>A]	260207	27	18	HM	CF at 0.5 m	Clear	Clear	Clear	12	12	Bony spicu les, ERM
IRD27	Yes	*CDHR1*<br>NM_033100.3<br>[c.338delG]	270502	26	16	CF at 2m	0.1	Clear	Clear	Clear	Soft	Soft	Abnormal foveal reflection
IRD28	Yes	*CRB1*<br>NM_201253.2<br>[c.1733T>A]	280413	48	NA	CF clos ely	HM	Clear	No L.O	No L.O	18	18	Severe pigmentary changes, RP triad, and asteroid hyalosis
IRD29	Yes	-	290407	30	7	0.5	NLP	Clear	PSCC	PSCC	Soft	Soft	RP triad
IRD30	Yes	-	300505	49	5	0.2	NLP	Clear	Blue dot cataract	Blue dot cataract	10	10	Bony spicules, pale optic disk, ischemic retina, diffuse white deposits, and atrophic hole
IRD31	Yes	*TULP1*<br>NM_003322.3<br>[c.1087G>A]	310404	48	NA	0.3	0.1	Clear	Early PSCC	Early PSCC	15	15	Bony spicules

IRD32	No	-	320404	14	NA	0.7	0.6	Clear	Clear	Clear	9	10	RP triad
			320301	44	NA	0.05	0.1	Clear	Early L.O	PCIOL	13	9	RP triad
			320302	40	NA	0.05	0.05	Clear	PSCC	PSCC	13	13	RP triad
IRD33	Yes	*CRB1* NM_201253.2 [c.2308G>A]	330401	17	3	NA	NA	NA	NA	NA	NA	NA	NA
			330402	11	3	NA	NA	NA	NA	NA	NA	NA	NA
IRD34	No	-	340202	4	1	CS M	CS M	Clear	Clear	Clear	Soft	Soft	Bony spicules
IRD35	No	*CERKL* NM_00103031 1.2 [c.847C>T/ c.481+1G>A]	350208	37	18	LP	LP	Apical scar	L.O	L.O	Soft	Soft	Peripheral Bony spicules
IRD36	No	*CLRN1* NM_00119579 4.1 [c.323T>C]	360203	41	36	0.3	CF at 2m	Clear	PSCC	PSCC	Soft	Soft	RP triad
IRD37	No	*USH2A* NM_206933.2 [c.8917_8918de l]	370403	53	NA	0.2	0.2	Clear	PCIOL	PCIOL	Soft	Soft	RP triad
IRD38	Yes	*IMPDH1* **NM_000883.3** [c.835T>G]	380401	28	3	0.1	0.4	Clear	Trace NS	Trace NS	14	14	RP triad
IRD39	Yes	*CRB1* NM_201253.2 [c.1844G>T]	390404	19	4	CF at 1m	CF at 1m	Clear	No L.O	No L.O	Soft	Soft	RP triad
IRD40	Yes	-	400502	26	12	0.5	0.1	Clear	No L.O	No L.O	Soft	Soft	RP triad
			400503	27	12	CF at 0.5 m	CF clos ely	Clear	No L.O	No L.O	Soft	Soft	RP triad
IRD41	Yes	*C8orf37* **NM_177965.3** [c.155+1G>A]	410502	35	8	0.05	CF at 2.75m	Clear	Clear	Clear	12	11	RP triad and PPA
IRD42	No	-	420204	18	NA	HM	HM	Clear	Post polar	Clear	Soft	Soft	RP triad
IRD43	No	-	430211	51	NA	NLP	NLP	Clear	Clear	Clear	Soft	Soft	RP triad
IRD44	No	-	440210	51	40	CF at 1m	NLP	Clear	Clear	Clear	15	13	Scattered bony spicules, pigmentary changes, and PVD

IRD45	Yes		450404	33	15	NA	NA	NA	NA	NA	NA	NA	NA
			450405	27	10	0.1	0.3	Clear	PSCC+2	PSCC+1	NA	NA	RP triad and ERM
			450406	29	12	0.1	0.3	Clear	PSCC+2	PSCC+1	NA	NA	RP triad and ERM
IRD46	Yes	*MERTK* **NM_006343.2** [c.2362G>A]	460401	36	5	CF at 1.25m	CF at 0.75m	Clear	ASCC+PSCC	ASCC+ PSCC	12	12	RP triad
IRD47	Yes	*CRB1* [c.3307G>A]	470401	55	6	LP	LP	Clear	Clear	Clear	Soft	Soft	Bony spicules
IRD48	Yes	*ABCA4* NM_000350.2 [c.5460+1G>A]	480404	19	8	CF at 2m	CF at 2m	Dry	ASCC+PSCC	ASCC+PSCC	NA	NA	Attenuated blood vessels, macular and perimacular bony spicules
			480406	23	10	CF at 2m	CF at 2m	Dry	No L.O	No L.O	Soft	Soft	Attenuated blood vessels, macular and perimacular bony spicules
IRD49	Yes	*AHI1* NM_017651.4 [c.2335G>A]	490406	12	NA	0.2	0.05	Clear	Clear	Clear	Soft	Soft	RP triad
IRD50	No	*CEP290* NM_025114.3 [c.4393C>T/ c.7089A>T]	500204	26	NA	CF at 0.5 m	0.2	Clear	PSCC	No L.O	16	16	RP triad and atrophic macula
IRD52	No	-	520303	57	NA	HM	LP	Clear	L.O	L.O	Soft	Soft	RP triad
IRD53	No	-	530303	20	NA	0.5	0.5	Clear	No L.O	No L.O	13	17	RP triad
			530304	19	NA	0.5	0.5	Clear	No L.O	No L.O	13	13	RP triad
IRD54	Yes	-	540504	9	2	CF at 1 m	CF at 1.5 m	Clear	No L.O	No L.O	Soft	Soft	Attenuated blood vessels
			540505	16	2	HM	CF at 0.5 m	Clear	Clear	Clear	Soft	Soft	RP triad
IRD55	Yes	*RDH12* NM_152443.2 [c.821T>C]	550402	15	NA	NA	NA	NA	NA	NA	NA	NA	RP triad
			550403	11	NA	NA	NA	NA	NA	NA	NA	NA	RP triad

			550404	16	NA	NA	NA	NA	NA	NA	NA	NA	RP triad
IRD56	Yes	*CNGB1* **NM_001297.4** [c.2662G>A]	560403	32	NA	LP	HM	Clear	No L.O	No L.O	Soft	Soft	RP triad
			560404	34	NA	HM	HM	Clear	PSCC	PSCC	12	12	RP triad

Table S6. Candidate variants with low evidence of pathogenicity identified in Jordanian IRD cohort.

Family ID	Gene	Variant coordinate hg19	HGVS variant nomenclature	dbSNP ID	gnomAD	Zygosity	Segregation analysis		ClinVar*	*In-silico* predictions SIFT, PP, MT	Reference
							Affected	Unaffected			
**IRD13**	***ERCC6***	**chr10:50724268**	**NM_170753.3: c.893G>A; p.(Trp298*)**	**rs1379548348**	**0.00005437**	**Het**	**1/2**	**1/3**		**T, B**	**Novel**
**IRD15**	***PRSS56***	**chr2:233387508**	**NM_001195129.1: c.643G>C; p.(Glu215Gln)**	**rs1220306609**	**0.00002299**	**Hom**	**1/2**	**0/2**		**T, B**	**Novel**
**IRD23**	***KCNJ13***	**chr2:233636036**	**NM_002242.4: c.37C>G; p.(Leu13Val)**	**rs1262517271**	**NA**	**Het**	**1/1**	**2/3**		**T, B, DC**	**Novel**
**IRD29**	***RGR***	**chr10:86008710**	**NM_002921.3: c.281C>T; p.(Ala94Val)**		**NA**	**Het**	**1/1**	**2/3**		**T, B, DC**	**Novel**
**IRD30**	***PITPNM3***	**chr17:6381326**	**NM_031220.3: c.869G>A; p.(Ser290Asn)**		**NA**	**Het**	**1/1**	**1/3**		**D, P, DC**	**Novel**
**IRD30**	***HMCN1***	**chr1:186014831**	**NM_031935.2: c.6316A>G; p.(Ile2106Val)**	**rs149970975**	**0.0001201**	**Het**	**1/1**	**1/3**		**D, P, DC**	**Novel**
IRD32	*OPN1SW*	chr7:128415610	NM_001708.2: c.235G>A; p.(Gly79Arg)	rs104894031	0.001592	Het	2/3	1/1		D, P, A	[1]

IRD34	*ROM1*	chr11:62381084	NM_000327.3: c.339delG; p.(Leu114Serfs*8)	rs760155276	NA	Het	1/1	2/2		D, D, DC	[2]
**IRD34**	***CAPN5***	**chr11:76830146**	**NM_004055.4: c.1238C>T; p.(Thr413Met)**	**rs370528920**	**0.0004174**	**Het**	**1/1**	**1/2**		**T, B, DC**	**Novel**
**IRD42**	***COL2A1***	**chr12:48371204**	**NM_001844.4: c.3172C>T; p.(Arg1058Cys)**	**rs148350640**	**0.00008038**	**Het**	**1/1**	**1/3**		**D, D, DC**	**Novel**
**IRD42**	***COL9A1***	**chr6:70942400**	**NM_001851.4: c.2389C>T; p.(Pro797Ser)**	**rs762152784**	**0.00003272**	**Het**	**1/1**	**3/3**		**D, D, DC**	**Novel**
IRD42	*PAX2*	chr10:102568941	NM_003990.4: c.936C>G; p.(Asn312Lys)	rs199724772	0.003375	Het	1/1	1/3	LB	T, B, DC	ClinVar
**IRD43**	***IMPG2***	**chr3:100961735**	**NM_016247.3: c.2819A>G; p.(Gln940Arg)**	**rs373286123**	**0.0002604**	**Het**	**1/1**	**1/1**		**T, D, DC**	**Novel**
IRD45	*ADGRV1*	chr5:89992918	NM_032119.3: c.8110A>T; p.(Ile2704Phe)	rs376318779	0.001685	Hom	2/3	0/3	VUS	D, P, DC	ClinVar

* At least one star status. Table S6 Abbreviations: PP: PolyPhen, MT: MutationTaster, DC: Disease Causing, A: Disease causing Automatic, D: damaging, N: neutral, B: benign, T: tolerated, NA: not available, VUS: variant of unknown significance; P, pathogenic; LP, likely pathogenic, Het: heterozygous, Hom: homozygous.

1. Weitz, C.J.; Miyake, Y.; Shinzato, K.; Montag, E.; Zrenner, E.; Went, L.N.; Nathans, J. Human tritanopia associated with two amino acid substitutions in the blue-sensitive opsin. *Am J Hum Genet* **1992**, *50*, 498-507.
2. Boulanger-Scemama, E.; El Shamieh, S.; Démontant, V.; Condroyer, C.; Antonio, A.; Michiels, C.; Boyard, F.; Saraiva, J.P.; Letexier, M.; Souied, E.; et al. Next-generation sequencing applied to a large French cone and cone-rod dystrophy cohort: mutation spectrum and new genotype-phenotype correlation. *Orphanet J Rare Dis* **2015**, *10*, 85, doi:10.1186/s13023-015-0300-3.
